# Bone Morphogenetic Protein-2 Induces Non-Canonical Inflammatory and Oxidative Pathways in Human Retinal Endothelial Cells

**DOI:** 10.3389/fimmu.2020.568795

**Published:** 2021-01-29

**Authors:** Mohamed Al-Shabrawey, Khaled Hussein, Fang Wang, Ming Wan, Khaled Elmasry, Nehal Elsherbiny, Heba Saleh, Paul B. Yu, Amany Tawfik, Ahmed S. Ibrahim

**Affiliations:** ^1^ Department of Oral Biology and Diagnostic Sciences, Dental College of Georgia, Augusta University, Augusta, GA, United States; ^2^ Department of Cellular Biology and Anatomy, Medical College of Georgia, Augusta University, Augusta, GA, United States; ^3^ Department of Ophthalmology and Culver Vision Discovery Institute, Medical College of Georgia, Augusta University, Augusta, GA, United States; ^4^ Department of Anatomy, Mansoura Faculty of Medicine, Mansoura University, Mansoura, Egypt; ^5^ Department of Medicine and Surgery, Oral and Dental Research Division, National Research Centre, Cairo, Egypt; ^6^ Department of Traditional Chinese Medicine, School of Medicine, Jianghan University, Wuhan, China; ^7^ Department of Biochemistry, Faculty of Pharmacy, Mansoura University, Mansoura, Egypt; ^8^ Division of Cardiovascular Medicine, Department of Medicine, Brigham and Women’s Hospital and Harvard Medical School, Boston, MA, United States; ^9^ Department of Ophthalmology, Visual, and Anatomical Sciences, Department of Pharmacology, Wayne State University, Detroit, MI, United States

**Keywords:** VEGF - vascular endothelial growth factor, smad, diabetic retinopathy (DR), bone morphogenetic protein - 2, p38 MAKP signaling

## Abstract

The mechanisms of diabetic retinopathy (DR), are not yet fully understood. We previously demonstrated an upregulation of retinal bone morphogenetic protein-2 (BMP2) in experimental diabetes and in retinas of diabetic human subjects. The purpose of current study was to investigate the role of non-canonical inflammatory pathway in BMP2-induced retinal endothelial cell (REC) barrier dysfunction. For this purpose, we used RT-PCR and western blotting to evaluate the levels of BMP2 signaling components (BMP2, BMP4, BMP receptors), VEGF, phosphorylated p38 MAPK and NFκB, and oxidative stress markers in cultured human retinal endothelial cells (HRECs) subjected to BMP2 (50ng/ml) for up to 24 h. Also, effect of high glucose (HG, 30mM D-glucose) on the expression of BMP2 and its downstream genes was examined in HRECs. H2-DCF is a fluorogenic dye that measures the levels of cellular reactive oxygen species (ROS) was used to measure the pro-oxidative effect of BMP2. Moreover, we evaluated the effect of inhibiting p38 and VEGF signaling on BMP2-induced HRECs barrier dysfunction by measuring the trans-endothelial cell electrical resistance (TER) using electric cell-substrate impedance sensing (ECIS). We also tested the effect of HG on the integrity of HRECs barrier in the presence or absence of inhibitors of BMP2 signaling. Our data reveals that BMP2 and high glucose upregulates BMP components of the BMP signaling pathway (SMAD effectors, BMP receptors, and TGFβ ligand itself) and induces phosphorylation of p38 MAPK and NFκB with nuclear translocation of NFκB. Inhibition of p38 or NFκB attenuated BMP2-induced VEGF expression and barrier dysfunction in HRECs. Also, inhibition of VEGFR2 attenuated BMP2-induced barrier dysfunction. Moreover, BMP2 induces generation of ROS and endothelial nitric oxide synthase (eNOS) expression and activity in HRECs. Finally, HG upregulated BMP2 and its downstream genes (SMAD, BMP4, ALKs, and TGF-β) in HRECs and BMP2 inhibitors attenuated HG-induced HRECs barrier dysfunction. Our results suggest that in addition to the regular canonical SMAD signaling BMP2 induces non-canonical inflammatory pathway in HRECs *via* activation of p38/NFκB pathway that causes the upregulation of VEGF and the disruption of HRECs. Inhibition of BMP2 signaling is a potential therapeutic intervention to preserve endothelial cell barrier function in DR.

## Introduction

Diabetic retinopathy (DR), a common microvascular complication of diabetes mellitus is considered the most common cause of blindness among working-age people in the world with deleterious socioeconomic impacts (1–3). Loss of blood-retinal barrier (BRB) function is a key stage in the development of DR leading to diabetic macular edema (DME) and subsequently loss of vision (4, 5). The cellular components of inner BRB include endothelial cells, pericytes, and glial cells (6, 7). Therefore, integrity of retinal endothelial cell barrier is essential to preserve normal function of BRB. Although current therapies including, laser photocoagulation, anti-vascular endothelial growth factor (VEGF), and corticosteroid demonstrated significant efficacy in treating DR and prevention of its progression, they are still limited by their significant side effects incomplete ability to eliminate the risk of blindness, and mostly applied in late stages of the disease (8–10). Therefore, there is a great demand for the development of novel independent or complementary therapeutic approaches that target primarily the early insults that lead to the development of irreversible visual loss.

Bone morphogenetic proteins (BMPs) are group of cytokines that belong to the transforming growth factors-β superfamily and were initially discovered and named for their ability to induce bone and cartilage formation (11, 12), however further studies of BMPs signaling pathway resulted in greater understanding of their crucial role. Nowadays they are increasingly recognized as multifunctional regulators of angiogenesis, tissue homeostasis and tumorigenesis, with evidence of activation of BMP signaling activity in different disease contexts (13–16). Among various members of BMPs, BMP2 has been the most studied and clinically relevant member. BMP2 was reported as an osteo-inductive cytokine that induces the entire cascade of cartilage and bone formation. Moreover, many studies linked BMP2 to various organs development including lung, heart, and central nervous system (17). However, BMP2 has been shown to have a pathological role associated with the development of vascular inflammation and angiogenesis (18, 19). This was confirmed by discovering the BMP endothelial cell precursor derived regulator (BMPER), a negative regulator of BMPs signaling. BMPER was shown to protect against vascular inflammation and preserve normal retinal vascular homeostasis *via* suppression of BMP signaling pathway (19, 20). In addition to BMPER, BMP2 is regulated by various extracellular BMP-regulating factors such as noggin, chordin, and gremlin (21–25).

Similar to other TGF-β family members, BMPs act through binding to a tetraheteromeric serine threonine kinase receptor complex (26). BMP receptors (BMPRs) compose of two BMPR type 1 receptors (BMPR1s) and two BMP type 2 receptors (BMPR2s). The biological effect of BMP2 is determined by its interaction with BMP type 1 receptor, since BMPR2 is a low affinity receptor (27, 28). There are four BMP type 1 receptor, Alk1/*Acvrl1*, Alk2/*Acvr1*, Alk3/*Bmpr1a*, and Alk6/*Bmpr1b* (28, 29). Affinity of various BMPs determines their different effects on endothelial cell function. For example, BMP9/10, which have an anti-angiogenic effect, have higher affinity to ALK1 whose deletion causes exuberant angiogenesis, suggesting that ALK1 regulates the angiostatic effect of BMP9/10 in endothelial cells (30, 31). On the other hand, ALK2, ALK3, and ALK6 that bind to BMP2, BMP4, and BMP6 are suggested to regulate their angiogenic signaling. Global deletion of these receptors is lethal and generally there is lack of understanding of their role in endothelial cell function in health and diseases such as diabetes and its microvascular complications as diabetic retinopathy (DR). Binding of BMPs to type I and II BMP receptors activates and phosphorylates receptor-regulated SMAD (R-SMAD) proteins (Smad 1, 5, and 9). Activated Smad 1, 5, and 9 proteins form a complex with co-Smad 4 and then translocate into the nucleus with subsequent interaction with other transcription factors to induce gene expression (32).

Our previous study was the first to demonstrate the upregulation of BMP2 in retinas of diabetic human subjects and in experimental mice as well as HRECs subjected to high glucose (HG). BMP2 also increased permeability, leukostasis, and inflammatory cytokines in HREC (33). However, the lack of understanding the molecular mechanism by which BMP2 induces REC dysfunction is a critical barrier in proposing it as a therapeutic target to treat DR. Thus, the aim of the current study is to delineate the molecular mechanisms by which BMP2 induces retinal endothelial cell barrier dysfunction which is essential for development of diabetic macular edema and pathological retinal neovascularization.

## Materials and Methods

### Experimental Animals

We used streptozotocin (STZ)-injected C57BL/6J mice and Ins2Akita mice (C57BL/6-Ins2Akita/J Stock No: 003548/Akita, Jackson Laboratories) as experimental mouse models of type1 diabetes mellitus (DM), while homozygote db/db (BKS/db−/−, Jackson Laboratories) mice were used as mouse model for type 2 DM that develops spontaneous glucose intolerance and hyperglycemia at 4–8 weeks. Heterozygote mice (db/+), which usually show normal body weight, blood glucose, and plasma insulin, but have increased metabolic efficiency were also used for comparison (34). For STZ injected mice, 6–8 weeks old mice were injected with STZ (55mg/kg, intra-peritoneal) for 3 consecutive days as previously described (35). Mice with plasma glucose level exceeding 250 mg/dl were considered diabetic. All experimental procedures were performed in accordance with the established guidelines of Association of Research in Vision and Ophthalmology statement for the Use of Animals in Ophthalmic and Vision Research, and were approved by the Institutional Animal Care and Use Committee (IACUC) of Augusta University. Blood was collected from cardiac puncture in heparinized coated tubes and the plasma was separated by centrifugation at 1,500 rpm for 20 min.

### Cell Culture

HRECs were obtained from Cell Systems (Kirkland, WA) and grown to 80–90% confluence in Endothelial Basal Medium-2 (EBM-2, LONZA). Cells were then serum starved for 24 h before treatment and throughout the whole experiment (1–5 days). HRECs were treated with rhBMP2 (50 ng/ml) with or without inhibitors of BMP signaling: Noggin (200ng/ml): an inhibitor of BMPs; LDN-193189 (LDN1, 200nM), a selective inhibitor of ALK2 and ALK3; LDN-212854 (LDN2, 200nM), an inhibitor of ALK2 with substantially weaker effects on ALK1 and ALK3. LDN1 and LDN2 were synthesized as previously described (36, 37). Moreover, HRECs were subjected to BMP2 treatment for 1–5 days in the presence or absence of various inhibitors such as inhibitors of VEGFR2 (SU5416, 10 μM) or p38 (SB202190), tyrosine kinase (genistein, 50µM), or ERK (U0126, 10µM) followed by assessment of the changes in barrier function and VEGF expression. Transfection of HRECs was done as previously described (35, 38) to silence Smad1 using SignalSilence^®^ Smad1 small interfering RNA (siRNA) (catalog # 6223, Cell Signaling Technology). The high glucose conditioned media (DG-CM) were prepared by incubating HRECs with high glucose (25 mM) for 5 days. Control conditioned media (LG-CM) were prepared by incubating cells with medium containing L-glucose (19.5 mM L-glucose, 5.5mM D-glucose; final concentration of 25 mM) for 5 days. Conditioned media were collected and concentrated 10 fold by spin-filtration (10 kDa cutoff, Millipore UFV4BK10) and added to the new HREC cultures.

### Electric Cell-Substrate Impedance Sensing Method

Normalized transcellular electrical resistance (TER) was measured by electric cell-substrate impedance sensing [ECIS^®^Zθ (theta)] instrument (Applied Biophysics Inc, Troy, NY, USA) as previously described (35, 38, 39). Briefly, HRECs were grown in 96-wells electrode arrays (catalog # 96W20idf PET, Applied Biophysics Inc.) coated with 100 µM cysteine and 0.02% gelatin. After confluence, cells were serum starved for 24 h and then treated with various treatments (BMP2 in the presence or absence of various inhibitors as above). For high glucose treatment, we used conditioned media (CM) that were collected from NG or HG-treated HRECs for 5 days. Fresh HRECs, then were subjected to these CM with or without various inhibitors (LDN1, LDN2, or noggin). TER was measured independently in each well over the time course of the experiment (4–5 days). Resistance values were normalized as the ratio of measured resistance to baseline resistance (normalized resistance) and plotted as a function of time.

### ELISA for BMP2 and VEGF

Levels of BMP2 in mouse plasma and in the cultured media collected from HRECs subjected to VEGF (30 ng/ml) for 24 h were quantified using ELISA kits following the manufacturer’s protocol (R&D Systems, Minneapolis, MN). In brief, 100 μl of Assay Diluent RD1-19 was first added to each well followed by addition of 50 μl of the standards and samples. Plates were incubated at room temperature for 2 h. Then, wells were washed three times using 400 μl wash buffer for each well. Two hundred microliters of Monoclonal antibody specific for BMP-2 was then added per well and kept at room temperature for 2 h. The wash step was repeated. Two hundred microliters of substrate solution was added to each well, protected from light and incubated at room temperature for 30 min. To stop the reaction, stop solution was finally added (50 μl) to each well. Detection of the optical density was done within 30 min, using a microplate reader at wavelength 450 nm with correction at 540 nm. Similar ELISA protocol was used to measure the levels of VEGF in the in the cultured media collected from HRECs that were subjected to BMP2 (50ng/ml) for 24 h in the presence or absence of p38 or NFκB inhibitors SB202190 (10μM) and JSH-23 (20μM) respectively.

### Quantitative Real-Time RNA Polymerase Chain Reaction Arrays

TaqMan arrays were used to measure messenger RNA (mRNA) levels of components of BMP2 signaling system in HRECs (Applied Biosystems, Foster City, CA). First, total RNA was extracted from HRECs subjected to rhBMP2, or HG using the RNeasy Mini Kit (Qiagen, Valencia, CA) according to manufacturer’s instructions. A high capacity cDNA synthesis kit (Applied Biosystems, Foster City, CA) was then used to synthesize cDNA. Q-PCR was performed using TaqMan Fast Advanced Master Mix Kit (Applied Biosystems, Foster City, CA) and PCR amplification was performed using Step One Plus Real-time PCR System (Applied Biosystem, Foster City, CA). The thermocycling program consisted of 50°C for 2 min and 95°C for 2 min, then 40 cycles at 95°C for 3 s, and 60°C for 3 min. Three replicates were run for each gene in each sample with the ready-made primer and probe sets in a 96-well plate. mRNA expression data was normalized to HPRT1 mRNA which has been used as the endogenous reference gene (housekeeping gene) as it does not exhibit significant expression changes between groups of samples. Calculations are based on the comparison of the distinct cycle determined by threshold values (Ct) at a constant level of fluorescence and the relative quantification of mRNA expression was calculated with the 2^-ΔΔCt method [Applied Biosystems User Bulletin N°2 (P/N 4303859)]. The data were normalized with respect to HPRT1 mRNA and relative to a calibrator sample. LG-conditioned media-treated HRECs were used as calibrators. ΔCt = (Ct target gene–Ct housekeeping gene). ΔΔCt = (ΔCt sample–ΔCt normal non-diabetic).

### Assessment of Effect of BMP2 Treatment on NF-κB and p-smad1/5/9 Nuclear Levels

HRECs treated with BMP2 (50ng/ml) for 30 min were harvested and the nuclear extract was prepared using nuclear extraction kit (Abcam Inc., Cambridge, MA, USA, ab113474). Western blot analysis was performed to detect the NF-κB (p65) and p-smad1/5/9 levels in the prepared nuclear extract. Briefly, equal amount of protein was loaded on gradient gel (4 to 20%, Pierce, Rockford, IL) and separated by sodium dodecyl sulfate polyacrylamide gel electrophoresis (SDS-PAGE). Thereafter, separated proteins were transferred into nitrocellulose membrane. The membrane was blocked using 5% BSA (Bio-Rad, Hercules, CA), washed, and then incubated overnight at 4°C with primary antibody for NF-κB (Cell Signaling, Danvers, MA, USA, 1:300) and the loading control histone deacetylase (HDAC) (Abcam Inc., Cambridge, MA, USA). The primary antibody reaction was then detected by membrane incubation with peroxidase-conjugated secondary antibody. The protein bands were then visualized using enhanced chemiluminescence (ECL) western blot detection system (Thermo Scientific, SC. USA) and the intensity of the immunoreactivity was measured using optical density analysis software (Image Lab, Bio-Rad Laboratories, USA).

### Immunofluorescence of smad4 and NF-κB

HRECs were stained with smad4 and NF-κB antibodies according to our previous procedure (40). Briefly, HRECs were fixed by using 2% paraformaldehyde followed by blockage in normal goat serum. Thereafter, HRECs were incubated with antibody against smad4 or NF-κB (Abcam, 1:100) overnight at 4°C followed by an incubation with Texas red labelled secondary antibody (1:500, Invitrogen, Eugene, OR). Finally, nuclei were stained with 4′,6 diamidino-2-phenylindole (DAPI) mounting medium (Vector Laboratories, Burlingame, CA), and images were taken with confocal microscopy (LSM 510; Carl Zeiss, Thornwood, NY).

### Assessment of Reactive Oxygen Species

DCF, the oxidation product of the reagent 2’,7’-dichlorofluorescin diacetate (H2DCFDA; Molecular Probes), was used as a marker of cellular ROS including superoxide (O2^−^), hydrogen peroxide (H2O2) and peroxynitrite (ONOO^−^) according to a previous procedure (41). Briefly, cells in 96-well plates were incubated in 50 μl of Earle’s balanced salt solution containing 5 µM H2DCFDA for 60 min and subjected to cellular DCF fluorescence measurement after BMP2 treatment (50 ng/ml for 24 h). Fluorescence was measured using a spectrofluorometer (BioTek Instruments) with excitation at 488 nm and emission at 530 nm. Similar procedures have been followed to measure the O2^−^ using the dihydroethidium (DHE, Sigma) which is more specific to superoxide generation than other ROS. The reaction intensity was measure at excitation 518 nm and emission 605 nm.

### Assessment of Nitric Oxide Generation

The assessment of nitric oxide (NO) was performed by measuring the total amount of nitrate and nitrite (which are the final products of NO) in HRECs after BMP2-treatment (50ng/ml for 18–24 h). The measurement was done by Fluorometric Assay Kit from Cayman Chemicals (Ann Arbor, MI, USA) in accordance with the manufacturer’s instructions. Briefly, HRECs were cultured in a 24-well black plate and then treated with BMP2 (50ng/ml) for 18–24 h. Thereafter, the samples were mixed with assay buffer and nitrate reductase mixture was added and incubated for 30 min to convert all nitrate to nitrite. To measure the total nitrite, 2,3 diaminonaphthalene reagent provided as acid solution was added followed by NaOH to enhance the detection of the fluorescent product. The fluorescence intensity was measured using spectrofluorometer at the excitation and emission wavelengths of 365 nm, 430 nm, respectively.

### Statistical Analysis

Differences among groups as represented by the mean ± SE were determined by the two-tailed *t* test or one way analysis of variance (ANOVA) followed by a *post hoc* Tukey’s test. Statistical significance among various groups was indicated by P value <0.05. For time-series studies, we used two ways ANOVA followed by a *post hoc* Tukey’s test for multiple comparisons.

## Results

### Effect of Experimental Diabetes on Circulating Levels of BMP2

**Figure 1:**
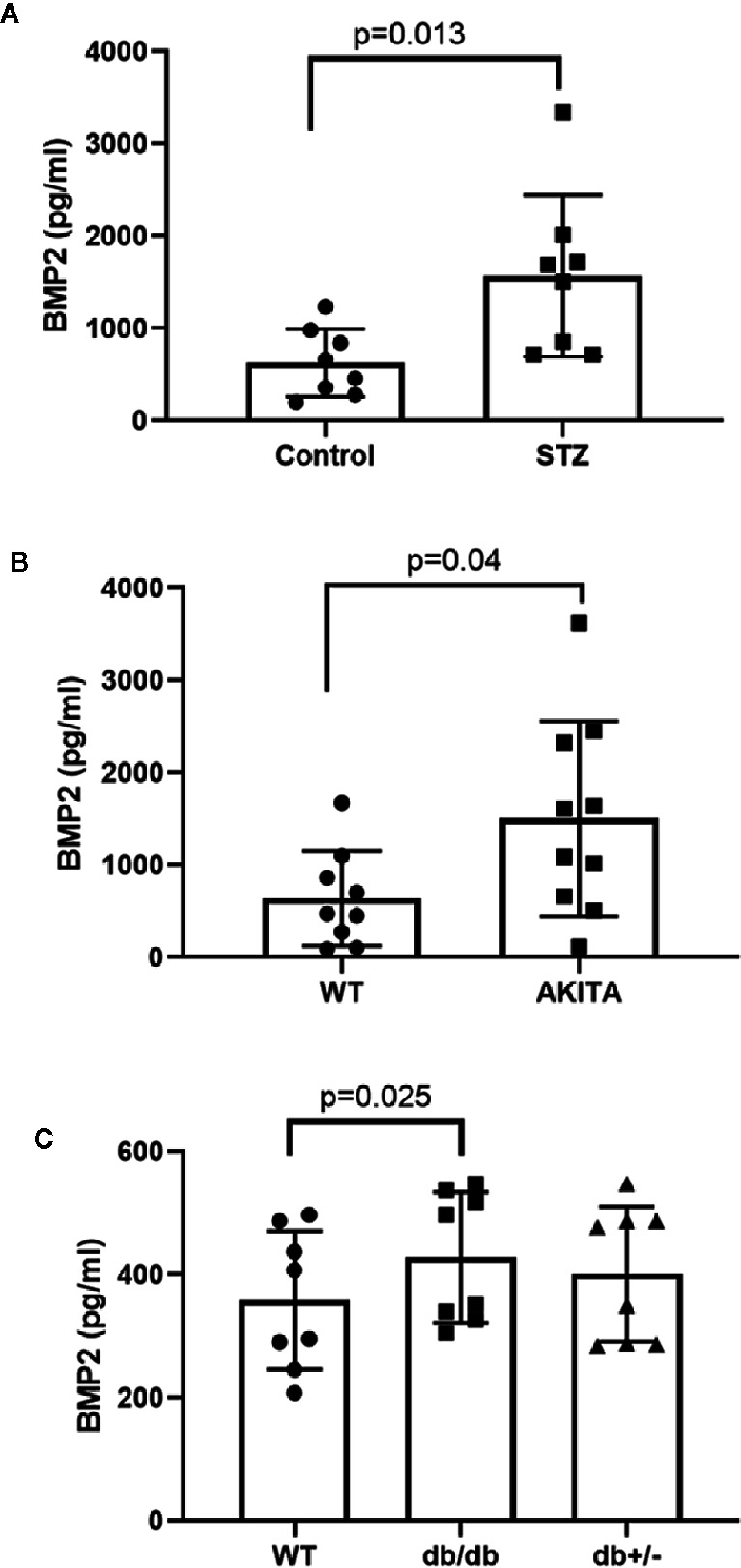
Measurement of circulating levels of BMP2 by ELISA. Unpaired t-test was used to compare the plasma levels of BMP2 in streptozotocin (STZ) or Akita diabetic mice compared to control. Data analysis showing significant increases STZ-diabetic (**A**, p value=0.013), and 12-months diabetic Akita mice (**B**, P=0.04). For multiple comparison, we used one way ANOVA followed by Tukey test to analyze the difference in plasma levels of BMP2 between db/db (6 week diabetic), db/+, and control. There was significant modest increase in the levels of plasma BMP2 in db/db compared to the control (**C**, p value = 0.025). There was no significant difference between db/db and db/+. n=8–10.

Our previous study showed a marked increase in retinal expression of BMP2 in experimental diabetes as well as in diabetic human (33). Here, we tested the changes in the levels of BMP2 in blood samples of experimental type 1 and type 2 diabetic mice (**Figure 1**). Plasma levels of BMP2 showed significant increases in experimental mouse models of type1 diabetes; the STZ (1,568 ± 309 *versus* 626 ± 130 pg/ml in control) and Akita mice (1503 ± 335 *versus* 638 ± 171 pg/ml in control). The increase in circulating BMP2 in STZ and Akita mice was reported after 6 and 12 months from the onset of diabetes, respectively. We also measured plasma levels of BMP2 in the 6-week diabetic db/db^−/−^ mouse model of type 2 diabetes in which there was significant increase (428 ± 37 *versus* 359 ± 40 pg/ml in control). Although we noticed a significant difference between db/db mice and its control, BMP2 levels in db/db were obviously lower than in STZ and Akita mice and no significant difference was noticed between the homozygote (db/db) and its heterozygote group (db/+) in which the mean of plasma BMP2 level is 401 ± 39 pg/ml.

### rhBMP2 and High Glucose Upregulate BMP Receptors and Activate Canonical smad Pathway in Human Retinal Endothelial Cells

**Figure 2:**
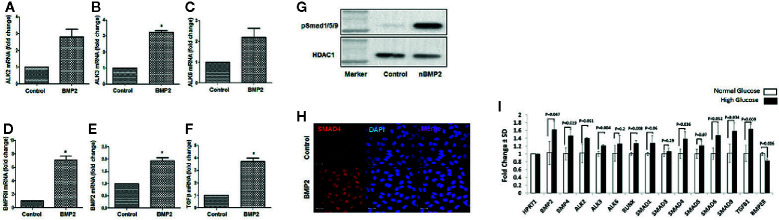
Effect of rhBMP2 or high glucose on BMP receptors and smad system. RT-PCR analysis of the messenger RNA (mRNA) of BMP receptors Alk2 **(A)**, ALK3 **(B)**, ALK6 **(C)** and BMPRII **(D)**, BMP2 **(E)** and TGFβ **(F)** in human retinal endothelial cells (HRECs) subjected to rhBMP2 (50ng/ml) for 24 h. There is a significant increase in the expression of BMP receptors ALK3 and BMPRII. There was also a marked increase ALK2 and ALK6 expression although this increase did not reach to significant levels. Also, BMP2 treatment induced significant increase in BMP2 and TGFβ mRNA expression. Western blotting of HRECs nuclear extract showing upregulation of nuclear psmad1/5/9 **(G)**. IF of smad4 showing marked increase in nuclear (blue) immunereactivity of smad 4 (red) by rhBMP2 **(H)**. n=6 (*P < 0.05). **(I)** The effect of hyperglycemia on various components of BMP2 signaling pathway. RT-PCR analysis of mRNA expression of various components of BMP2 signaling pathway in HRECs that were subjected to LG-CM or HG-CM treatment for 2 days.

Using RT-PCR we tested the effect of rhBMP2 on mRNA levels of BMPRs (ALK2, 3, 6, and BMPR2). Our data showed that BMP2 upregulates endothelial ALK3 and BMPRII (BMPR2) significantly. There was also increases in the expression levels of ALK2 and ALK6, although these increases did not reach significant levels. rhBMP2 also upregulated the mRNA levels of BMP2 and TGFβ compared to the control (**Figures 2A–F**). The increased expression of BMP2 in HRECs subjected to exogenous rhBMP2 may establish a link between increased blood levels of BMP2 and its upregulation in retina during diabetes.

Activation of the smad effectors represents the canonical pathway of the BMP2 signaling and plays an essential role in mediating various biological functions of BMP2 by first phosphorylation and assembly of smad1/5/9/4 complex then translocation to the nucleus and initiating a transcription activity of target genes. Here, we evaluated the effect of rhBMP2 on the nuclear translocation of BMP-responsive smads system in HRECs. We first analyzed the amount of phosphor-smad1/5/9 in the nuclear extract of HRECs that were subjected to treatment with rhBMP2 or its vehicle for 30 min using western blot. While p-smad1/5/9 was absent in the nuclear extract of the control HRECs, we noticed a higher amount in the rhBMP2-treated HRECs. In addition to smad1/5/9, smad 4 is also a part of smad complex that translocates to nucleus upon activation by BMP2. Therefore, we tested its translocation to the nucleus by immunofluorescence (IF) that showed a marked increase of smad4 nuclear immunoreactivity in rhBMP2-treated HRECs compared to the control (**Figures 2G, H**).

We also tested the effect of hyperglycemia on various components of BMP2 signaling pathway. RT-PCR analysis of mRNA expression of HRECs that were subjected to LG-CM or HG-CM treatment for 2 days showed significant increase in the expression of BMP2, BMP4, ALK3, smad9, smad4, and TGFβ mRNA by HG-CM compared to the osmotic control LG-CM (**Figure 2I**). We also noticed marked increases in ALK2, smad1, and SMAD5 mRNA although this increase did not reach statistical significance. Interestingly, HG-CM treatment induced a significant decrease in the expression of BMP binding endothelial regulator (BMPER) mRNA, a known negative regulator of BMP2 functions (42).

### BMP2 Induces P38/NFκB Non-Canonical Pathway in Human Retinal Endothelial Cells

**Figure 3:**
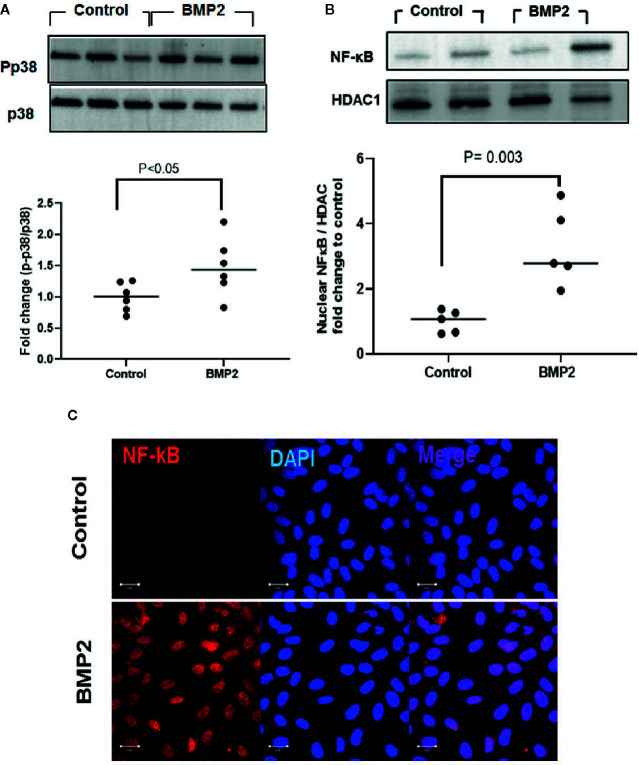
Western blot analysis of the effect of rhBMP2 on p38 and NFκB. Unpaired t-test was used to compare the effect of rhBMP2 on p-p38 and nuclear NFκB compared to control. Densitometry analysis showing significant increase in the levels of p-p38 and the nuclear NFκB in human retinal endothelial cells (HRECs) compared to the control (**A**, **B** respectively). IF **(C)** also showing marked increase in the nuclear (blue) immunoreactivity of NFκB (red) in HRECs subjected to rhBMP2 compared to the control. n=5–6.

Activation of p38 MAPK contributes to the biological functions of the BMP2/ALKs system and its role in DR has been well-established (43–47). We hypothesize that in addition to smad canonical pathway, p38 MAPK/NFκB signaling pathway also contributes to the permeability and inflammatory effects of BMP2 in the retina. In support to this hypothesis, analysis of the levels of phosphorylated p38 MAPK in HRECs showed 1.5 ± 0.2 fold increase by rhBMP2 compared to the vehicle treated cells (**Figure 3A**). The interaction between p38 MAPK and the transcription factor NFκB is implicated in the regulation of several inflammatory cytokines and oxidative stress mediators (48–51). Here, in addition to p38 MAPK activation, rhBMP2 also induced nuclear translocation of NFκB in HRECs as shown by western blot (3.3 ± 0.5) fold increase *vs*. control) and increased NFκB nuclear immunoreactivity by IF (**Figures 3B, C** respectively).

### Presence of a Positive Feedback Between BMP2 and VEGF Expression in Human Retinal Endothelial Cells

**Figure 4:**
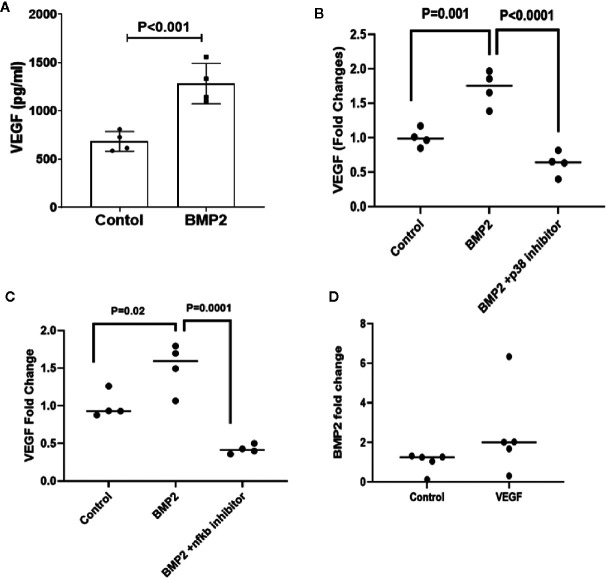
ELISA of VEGF and BMP levels lin human retinal endothelial cells (HRECs). One-way ANOVA followed by a *post hoc* Tukey’s test was used to compare the effect of BMP2 on VEGF levels in the presence or absence of p38 or NFκb inhibitors in cultured HRECs. Data analysis are showing significant increases in the expression of VEGF by BMP2 (50ng/ml) treatment for 24 h compared to the control **(A).** This increase is inhibited by p38 inhibitor (SB202190, 10μM) **(B)** or NFκB inhibitor (JSH23, 20 μM) **(C)**. Furthermore, ELISA of BMP2 levels in the condition medium of VEGF-treated HRECs is showing a significant increase by VEGF (30ng/ml for 24 h) compared to the control **(D)**. Analysis of the difference between control and VEGF treatment was done using unpaired t-test.

VEGF is a key player in the development of retinal endothelial cell dysfunction in DR and has been shown to be regulated by both smad canonical (52, 53) and non-canonical (54, 55) pathways. We tested whether there is an interaction between BMP2 and VEGF in retinal endothelial cells. While BMP2 induced significant increases in VEGF levels in HRECs (1,284± 209.9 pg/ml; mean ± SD) *versus* the control (686 ± 102.5 pg/ml; mean ± SD) (**Figure 4A**), inhibition of p38 or NFκB by SB202190 or JSH-2 respectively prevented this increase and reduced the levels of VEGF in HRECs to 0.6± 0.1 and 0.4 ± 0.03 fold, respectively, *versus* the control (**Figures 4B, C**).

Interestingly, treatment of HRECs with VEGF (30ng/ml) also upregulated BMP2 expression (2.5 ± 1.0 fold increase *vs*. control) (**Figure 4D**) suggesting presence of a positive feedback between BMP2 and VEGF in the retina, which could contribute to the development of vascular abnormalities in DR.

### Inhibition of smad Pathway Attenuates rhBMP2-Induced Human Retinal Endothelial Cells Barrier Dysfunction

**Figure 5:**
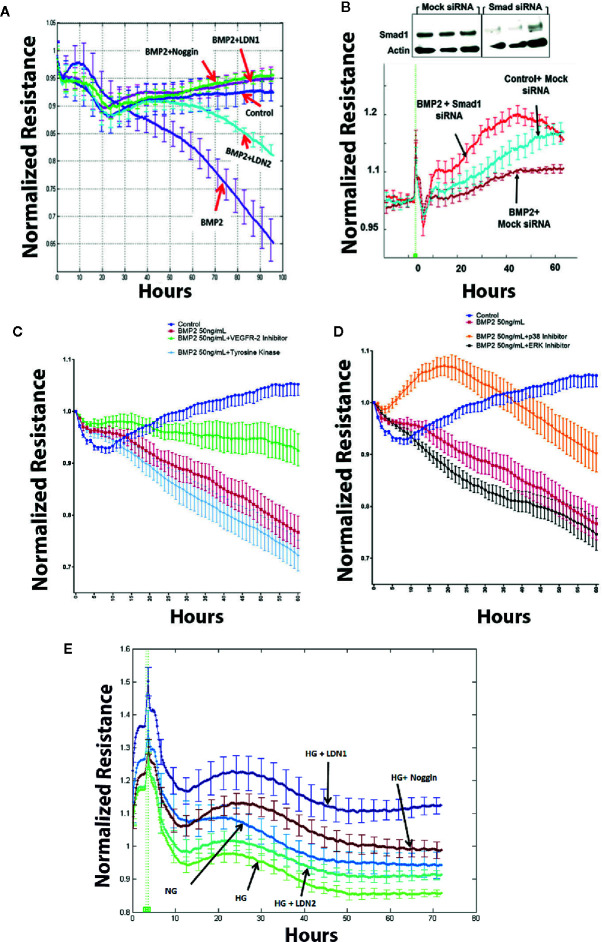
Effect of BMP2 on trans-endothelial electrical resistance (ECIS). ECIS analysis showing significant decreases in the trans-endothelial cell electrical resistance (TER) of human retinal endothelial cells (HRECs) treated with rhBMP2 (50ng/ml for 4–5 days). Effect of rhBMP2 was significantly attenuated by the inhibitors of BMP receptors, LDN1 and LDN2 as well as by the noggin that prevents BMP2 from binding its receptors **(A)**. Effect of rhBMP2 on the TER of HRECs was also attenuated by silencing smad1 using smad1 small interfering RNA (siRNA) compared to the mock siRNA **(B)**. Inhibition of VEGFR2 by SU5416, 10 μM **(C)**, or p38 by SB202190, 10 μM **(D)** showing significant attenuation of the rhBMP2-induced reduction of the TER in HRECs. However, inhibition of tyrosine kinase by G-6055 9Genistein 10µM **(C)** or MERK by U0126 10µM **(D)** did not prevent the effect of BMP2-induced changes in the TER of HRECs. Similarly, incubation of HRECs in CM of HG-treated HRECs induced significant decrease in TER over time and this effect was attenuated by noggin, LDN1, and LDN2. **(E)** Data points represent the mean of 4–6 wells at the same time point+SE. Two way ANOVA followed by a *post hoc* Tukey’s test. It was used to compare different treatments over time.

Transcellular electrical resistance (TER) reflects the integrity of cellular barrier function. To evaluate the extent to which BMPRs contribute to the permeability effect of BMP2, we tested the effect of specific inhibitors that target BMPRs or their binding to BMP2 on rhBMP2-induced changes in TER of HRECs using the ECIS. For example, we used noggin that blocks the binding of BMP2 to its receptors and LDN1 and LDN2 that block ALK2 and ALK3 receptors. Consistent with our previous study (33), rhBMP2 disrupted the barrier function of HRECs as shown by a significant decrease in the TER of HRECs. This disruptive effect was significantly attenuated by noggin, LDN1, and LDN2 (**Figure 5A**) supporting the role of BMPRs in mediating the permeability effect of BMP2 in HRECs. Silencing of smad1 (**Figure 5B**) also preserved HRECs barrier function under BMP2 treatment as compared to HRECs transfected with mock siRNA. These findings suggest a role for the smad pathway in BMP2-mediated HREC dysfunction.

### Inhibition of p38, or VEGF Signaling Attenuates BMP2-Induced Human Retinal Endothelial Cells Barrier Dysfunction

In addition to the effect of BMPRs inhibition, we also tested whether inhibition of non-canonical pathway represented by p38 and VEGF may impact the disruptive effect of rhBMP2 on HRECs TER using ECIS. Here, our experiments showed that effect of BMP2 on endothelial TER was significantly abrogated by inhibitors of VEGFR2 (SU5416, 10 μM) or p38 (SB202190) (**Figures 5C, D** respectively) On the other hand, inhibition of tyrosine kinase by genistein (50µM) compared to VEGFR2 inhibition or ERK by U0126 (10µM) compared to p38 MAPK inhibition did not prevent rhBMP2-induced reduction of TER.

### Inhibition of BMP2 Signaling Preserves Human Retinal Endothelial Cell Barrier Function Under Hyperglycemia

We also tested whether inhibition of BMP signaling offers protection against hyperglycemia-induced barrier dysfunction in HRECs. Barrier dysfunction usually occurs in HRECs after several days of HG treatment (>5 days) that affect the viability of HRECs cells and also the pH of the media. Therefore, we used CM of HRECs subjected to NG or HG for 5 days to treat new HRECs in the presence or absence of various BMP2 signaling inhibitors (LDN1, LDN2, or noggin). Our data showed that noggin LDN1and LDN2 preserve HRECs barrier function under HG condition (**Figure 5E**).

### BMP2 Induces Generation of Reactive Oxygen Species in Human Retinal Endothelial Cells

**Figure 6:**
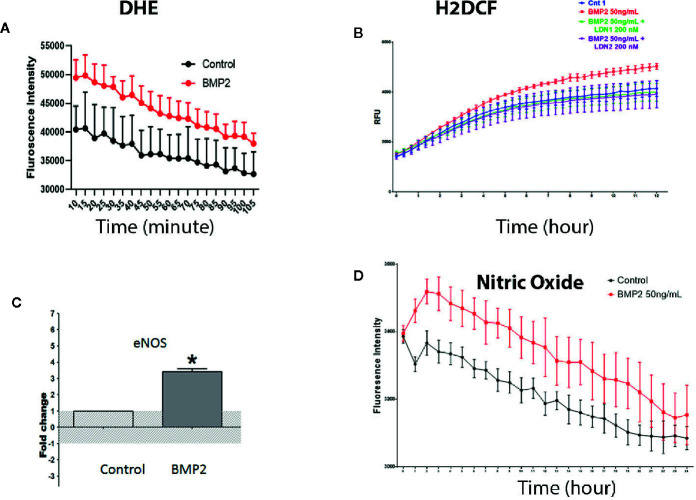
Measurement of intracellular reactive oxygen species by dihydroethidium or DHE **(A)** and H2-Dichlorofluorescine or DCF **(B)** showing significant increases in the superoxide and other ROS, respectively by BMP2. The increase in ROS was prevented by the pharmacological inhibitor of BMPR (LDN1). RT-PCR **(C)** showing significant increases in the levels of eNOS messenger RNA (mRNA) in the human retinal endothelial cells (HRECs) by BMP2. *p < 0.05. Finally, measurement of the eNOS activity by measuring nitric oxide (NO) generation using the fluorescence reaction **(D)** showing significant increases by the BMP2. Differences among groups as represented by the mean ± SD were determined by the two-tailed *t* test or two way ANOVA followed by a *post hoc* Tukey’s test.

Oxidative stress is a key player in mediating endothelial cell dysfunction in various diseases including diabetic retinopathy (38). Therefore, we tested the impact of rhBMP2 on HRECs redox status. Treatment of HRECs with rhBMP2 induced significant increase in the superoxide (O2^−^) generation as measured by DHE color reaction. DHE that only labels O2^−^ reached the peak after 10 min followed by time-dependent decrease although it stayed higher than control group (**Figure 6A**). We also assessed ROS generation using the H2-DCF a fluorogenic dye that measures the levels of various cellular ROS including O2^−^, H2O2, and ONOO^−^. Analysis of H2-DCF in HRECs showed a significant increase in ROS generation by rhBMP2. However, contrary to DHE (O2^−^), H2-DCF started to increase after 5 h from adding the rhBMP2 and continued to increase for several h compared to the control (**Figure 6B**). This suggests that while O2^−^ decreases over the time, total ROS that includes also H2O2 and ONOO^−^ in addition to O2^−^ continue to increase. This increase of ROS generation by rhBMP2 was significantly attenuated by ALK inhibitor LDN1.

We tested whether the consistent decrease of O2^−^ is attributed to generation of nitric oxide (NO) that reacts rapidly with O2^−^ to form ONOO^−^ and in turn increases H2-DCF reaction over time. For this purpose we tested the effect of rhBMP2 on the levels of eNOS and its activity (nitric oxide generation) as a known source of ROS particularly the ONOO^−^ by its reaction with O2^−^. RT-PCR of HRECs showed significant increase of eNOS mRNA by rhBMP2 compared to the control (**Figure 6C**) and this was associated with a significant increase in NO generation (**Figure 6D**). Interestingly, the increase in NO showed a similar pattern to O2^−^ generation in which it decreases over time although it consistently stays higher than the control (**Figure 6F**).

## Discussion

Our previous study (33) demonstrated upregulation of BMP2 in retina of diabetic human subjects and experimental diabetic mice and upregulation of inflammatory cytokines and leukostasis in cultured retinal endothelial cells subjected to BMP2 treatment. It was the first study to underscore BMP2 as a possible player in the development of microvascular dysfunction in DR. The current study extends to investigate in some details the mechanism by which BMP2 regulates retinal endothelial cell barrier function. The main findings of the current study are: 1) significant increase in the circulating BMP2 in experimental models of type 1 and type 2 diabetes, 2) induction of both canonical and non-canonical pathways in HRECs by BMP2, 3) attenuation of BMP2-induced permeability by inhibitors of canonical or non-canonical pathways, 4) induction of oxidative stress in HRECs subjected to BMP2.

The role of BMP signaling system in endothelial cell dysfunction has attracted several investigators who showed permeability and angiogenic potential of the activated BMP signaling (33, 51, 56, 57). However, the relationship between diabetes in general and its complications in particular and activated BMP signaling has not yet been established. Here, we provide the first evidence for the increase in the levels of circulating BMP2 in mouse model of type 1 (Akita and streptozotocin) and type2 diabetes (db/db mice) compared to their controls. Although there was significant increase in circulating BMP2 in db/db mice compared to their control wild type, this increase was modest compared to STZ and Akita models and was not significant compared to the heterozygote db/+ group. This raises a question regarding this difference between type 1 and type 2 diabetes and whether relative long exposure to hyperglycemia in STZ (6 months) and Akita (12 months) *versus* 10–12 weeks in db/db played a role in this differential effect of diabetes on plasma levels of BMP2. In addition, to the observed *in vivo* increase in BMP2 by hyperglycemia, treatment of HRECs with HG upregulated mRNA of BMP2 and BMP4 and their downstream effectors such as ALKs, SMADs, and TGFβ mimicking the effect of rhBMP2 treatment. Contrary, this increase of BMP2 signaling components by HG was associated with significant reduction in BMPER, a known negative regulator of BMP2 signaling (42). These data suggest that the balance between BMP2 signaling system and its negative regulators such as BMPER is important to maintain normal endothelial cell function.

Since our previous study showed upregulation of the retinal BMP2 (33) and the current one showing upregulation of the circulating BMP2 in diabetes, we suggest presence of a positive feedback between the circulating BMP2 and the retinal BMP2. Moreover, increases in the circulating BMP2 was reported here at early and late stage of diabetes, 6 week–12 months from onset of the experimental diabetes leading us to hypothesize that the circulating level of BMP2 could be a biomarker in the diabetic patients. This may correlate the circulating levels of BMP2 with development of diabetic complications such as DR. Further investigations are needed to prove this hypothesis in the diabetic patients.

BMP2 mediates its biological functions through activation of two independent or linked pathways, the smad (canonical) and p38 MAPK (noncanonical) pathways. Activation of both pathways is downstream from BMP2 binding to its receptors. The biological effect of BMP2 is determined by its interaction with BMPR1 particularly (ALK2, 3, and 6), since BMPR2 is a low affinity receptor (27, 28). Treatment of HRECs with rhBMP2 upregulated expression of BMPR mRNAs particularly ALK3 and BMPRII that showed significant increases. There was also marked increase in ALK2 and ALK6 although this increase did not reach significant levels. In agreement with previous reports (29, 58), endothelial ALK2, 3, and 6 as well as BMPRII are implicated in BMP2 signaling in retinal endothelial cells. Moreover, the disruptive effect of rhBMP2 on HRECs barrier function as demonstrated by a significant decrease of the TER was prevented by the BMPR inhibitors, noggin, LDN1, and LDN2. ALK2 and 3 have been shown to play important role in the proangiogenic effect of BMP2 and thus suggested to be selective target for the antiangiogenic therapy (51). A recent report has emphasized both ALK2, ALK3, and BMPRII in BMP2-induced retinal vascular development and targeted deletion of any of them in endothelial cells led to significant impairment of normal vascular development in retina (59).

We next investigated the impact of rhBMP2 on the nuclear translocation of smad complex which is required to induce the transcription activity of BMP2-dependent genes. Our experiments using western blot analysis of the HRECs nuclear extracts or immunofluorescence showed nuclear translocation of smad1/5/9 and smad4 respectively by rhBMP2 treatment. We then, asked if smad activation contributes to the permeability effect of BMP2. For this purpose, we tested the effect of smad1 silencing on rhBMP2-induced barrier dysfunction in HRECs. Smad1 siRNA preserved the TER of HRECs in the presence of rhBMP2 compared to the MOCK siRNA suggesting involvement of the smad system in the BMP2-induced retinal endothelial cell dysfunction.

In addition to the smad system, activation of the non-canonical pathway as represented by p38 MAPK/NFκB and dependent genes have also been shown to contribute to the biological effects of BMP2 (48–51). Therefore, we evaluated the hypothesis that this non-canonical pathway also contributes to the disruptive effect of rhBMP2 on the HRECs barrier function. To test this hypothesis, we examined the direct effect of rhBMP2 on the phosphorylation/activation levels of p38, nuclear translocation of NFκB as well as VEGF expression as one of the essential molecules that could be regulated by activated p38/NFκB signaling. Our experiments demonstrated a significant increase in the levels of phosphorylated-p38 MAPK and the nuclear translocation of NFκB. In addition, inhibition of p38 or NFκB significantly attenuated the rhBMP2-induced upregulation of VEGF, a key player in endothelial dysfunction in diabetic retinopathy. We next investigated if the activated non-canonical pathway contributes to rhBMP2-induced HRECs barrier dysfunction using specific inhibitors of p38 MAPK. Inhibition of p38 MAPK preserved the TER in the rhBMP2-treated HRECs compared to the ERK inhibitor. Our findings also suggest that p38/NFκB is implicated in BMP2-induced endothelial dysfunction probably *via* a VEGF-dependent mechanism. In agreement to our data, BMP receptor activation has been reported to selectively induce activation of the p38 mitogen-activated protein kinase (MAPK) in contrast to the ERK1/2 MAP kinases to promote tumor angiogenesis (60).

The potential role of VEGF signaling in BMP2-induced HRECs barrier dysfunction was demonstrated by the significant improvement of the HRECs TER by the VEGFR2 inhibition. VEGF has been shown to induce BMP2 expression in the microvascular endothelial cells (61). Our experiments using HRECs also showed an upregulation of BMP2 expression by VEGF treatment, suggesting presence of a positive feedback between VEGF and BMP2 in the retinal endothelial cells. We previously established a positive cross talk between BMP2 and VEGF to enhance the osteogenesis and angiogenesis in the critical size bone defect model (62, 63). VEGF and BMP2 have shown to have a synergistic effect in inducing osteogenesis, angiogenesis, and metastasis, therefore, administration or inhibition of both VEGF and BMP2 was suggested as a possible strategy to enhance bone healing or decreasing the incidence of cancer metastases respectively (56, 57). We previously showed that BMP2 also upregulate VEGF production in the retinal Müller cells. Current and previous data may establish an interesting relation between VEGF and BMP2 especially in diabetes in which increased circulating BMP2, retinal BMP2 and VEGF elicit positive feedback in both endothelial and Müller cells to contribute to the retinal microvascular dysfunction in diabetes. VEGF has been established as a downstream target from smad system and p38/NFκB (54, 55), thus we conclude that activation of the canonical and non-canonical pathways in HRECs by rhBMP2 converges at VEGF signaling to induce HRECs barrier dysfunction. In addition to studying the direct effect of rhBMP2 on HRECs barrier integrity in the presence or absence of its signaling inhibitors, we also evaluated the effect BMP2 signaling inhibitors on HG-induced barrier dysfunction. Our data showed preservation of HRECs barrier under hyperglycemic insult by BMP2 signaling inhibitors suggesting inhibition of BMP2 signaling as potential therapeutic intervention to protect blood-retinal barrier in diabetes.

We and others have reported the critical role of ROS such as O2^−^, ONOO^−^ and H2O2 in inducing endothelial permeability and angiogenesis in DR (64–66). We tested if the BMP2 elicits any effect on redox status of the retinal endothelial cells. Our data, showed significant increases of ROS generation in HRECs over an extended period of time by the rhBMP treatment. This effect was attenuated by pharmacological inhibition of BMPR using the LDN1 and LDN2. The peak of increased superoxide as measured by DHE staining was noticed after 10 min from adding rhBMP2 followed by a consistent decrease over time. Contrary the total amount of ROS generation (O2^−^, ONOO^−^, and H2O2) as measured by H2DCF continued to increase by the rhBMP2 treatment over time. This led us to test the effect of BMP2 on eNOS, a known source of ROS and NO. There was a significant increase in the mRNA of eNOS by rhBMP2. This was associated with a significant increase in NO generation that showed a similar pattern to superoxide generation in which NO continued to decrease over time. This led us to suggest that the generated O2^−^, and NO combine to generate ONOO^−^ and this was reflected on the consistent increase of the H2DCF reaction opposite to the consistent decrease in both superoxide and NO. Of note, increased oxidative stress leads to NOS uncoupling and NO-quenching by excess superoxide to form ONOO^−^ (67). This may explain the inverse relationship noticed between the total ROS and both O2^−^ and NO over an extended period.

In conclusion, the current study underscores the circulating BMP2 in addition to the retinal BMP2 as a potential player and probably a biomarker in diabetes complications such as DR. The study also characterizes the signaling mechanisms by which BMP2 may contribute to the retinal endothelial cell dysfunction especially hyperpermeability in DR. The underlying mechanism involves BMPRs and subsequent activation of canonical and non-canonical pathways. Downstream from these signaling pathways, VEGF and oxidative stress are probably contributing factors to BMP2-induced retinal endothelial cell dysfunction. Hence, the use of BMP signaling system inhibitors alone or in conjunction with anti-VEGF could be a novel approach to prevent the microvascular dysfunction in DR.

## Data Availability Statement

The original contributions presented in the study are included in the article/**Supplementary Material**. Further inquiries can be directed to the corresponding author.

## Ethics Statement

The animal study was reviewed and approved by the Institutional Animal Care and Use Committee (IACUC) of Augusta University.

## Author Contributions

All authors listed have made a substantial, direct, and intellectual contribution to the work and approved it for publication.

## Funding

This research was funded by the National Eye Institute grants R01EY030054-01A1 and R01EY023315 (MA-S); the American Heart Association Grant 18CDA34080403 (AI), NIH core grant P30EY004068 to the Department of Ophthalmology, Visual and Anatomical Sciences (OVAS), and a Research to Prevent Blindness unrestricted grant to the Department of OVAS, Wayne State University, Detroit, MI, USA; Chinese Cultural Bureau to (FW) and (MW); and Egyptian Cultural bureau (KH, NE, and KE). 1R01EY029751-01 to AT.

## Conflict of Interest

PY is a co-founder and scientific advisory board member for Keros Therapeutics, Inc., and holds stock and intellectual property (IP) as a result of his work done in the lab that has been licensed by the company. PY is also a consultant to Acceleron Pharma Inc. and holds IP related to technology stemming from work done in the lab that was licensed by that company.

The remaining authors declare that the research was conducted in the absence of any commercial or financial relationships that could be construed as a potential conflict of interest.

## Supplementary Material

Supplementary Data Sheet 1 Original Western blot for the p-p38 and NFkB data in HRECs subjected to BMP2 and BMP4.
