# Supplementary material for: Bone Morphogenetic Protein-2 Induces Non-Canonical Inflammatory and Oxidative Pathways in Human Retinal Endothelial Cells
Source: Front Immunol. 2021 Jan 29;11:568795. doi: 10.3389/fimmu.2020.568795 (PMC7878387; doi:10.3389/fimmu.2020.568795)
Supplement: Supplementary Data Sheet 1 — Original Western blot for the p-p38 and NFkB data in HRECs subjected to BMP2 and BMP4. [file DataSheet_1.pdf]

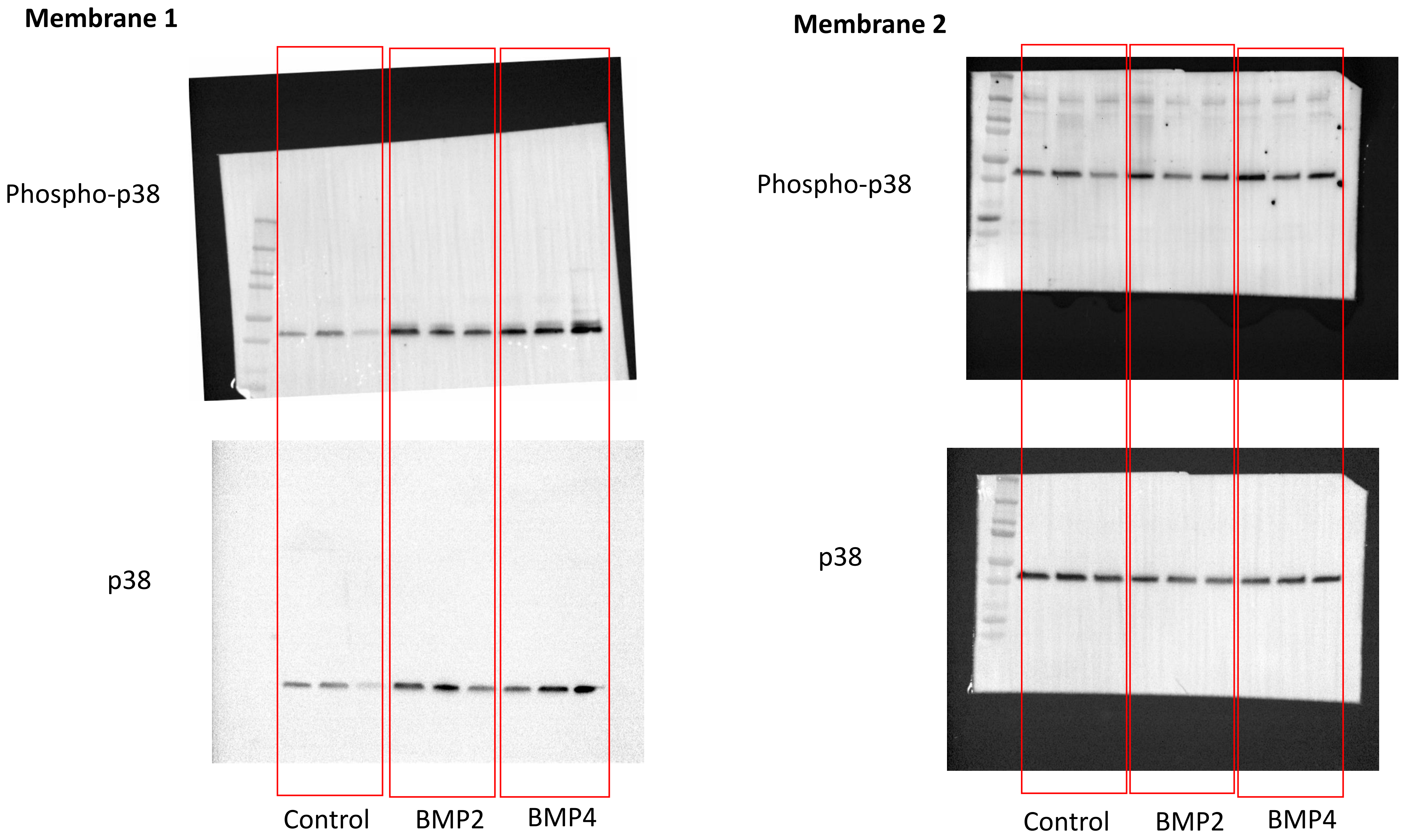
Membrane 1
Phospho-p38
p38
Control
BMP2
BMP4
Membrane 2
Phospho-p38
p38
Control
BMP2
BMP4

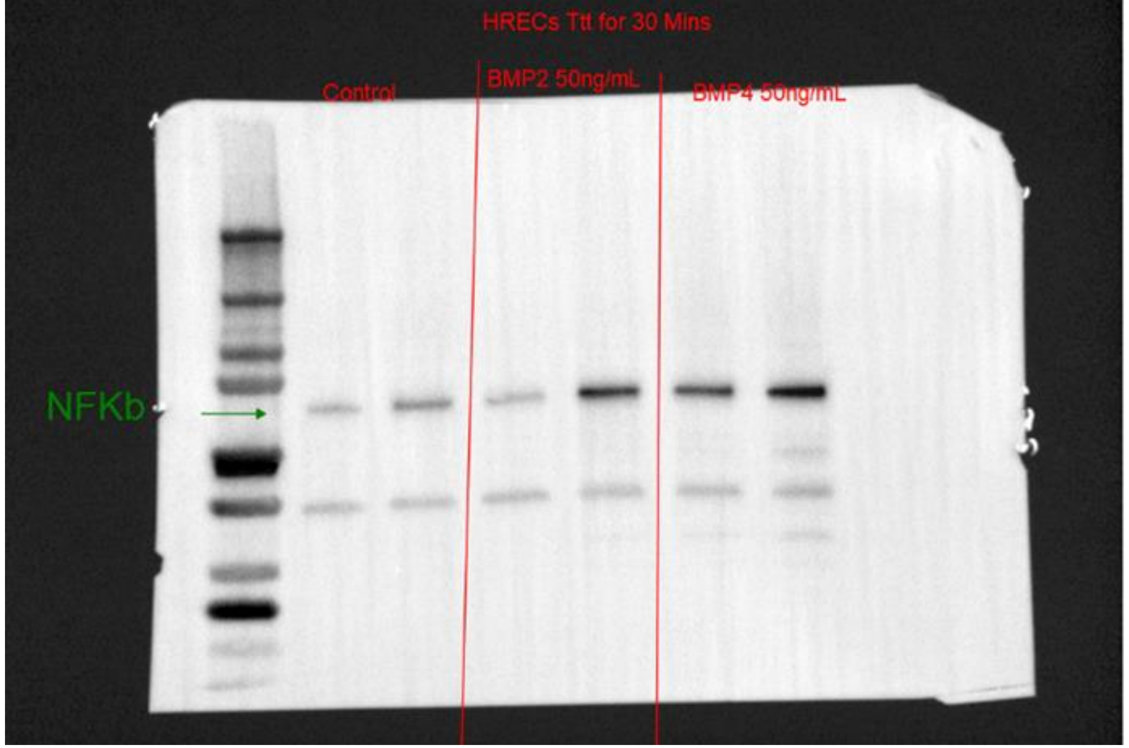
HRECs Ttt for 30 Mins
Control
BMP2 50ng/mL
BMP4 50ng/mL
NFKb

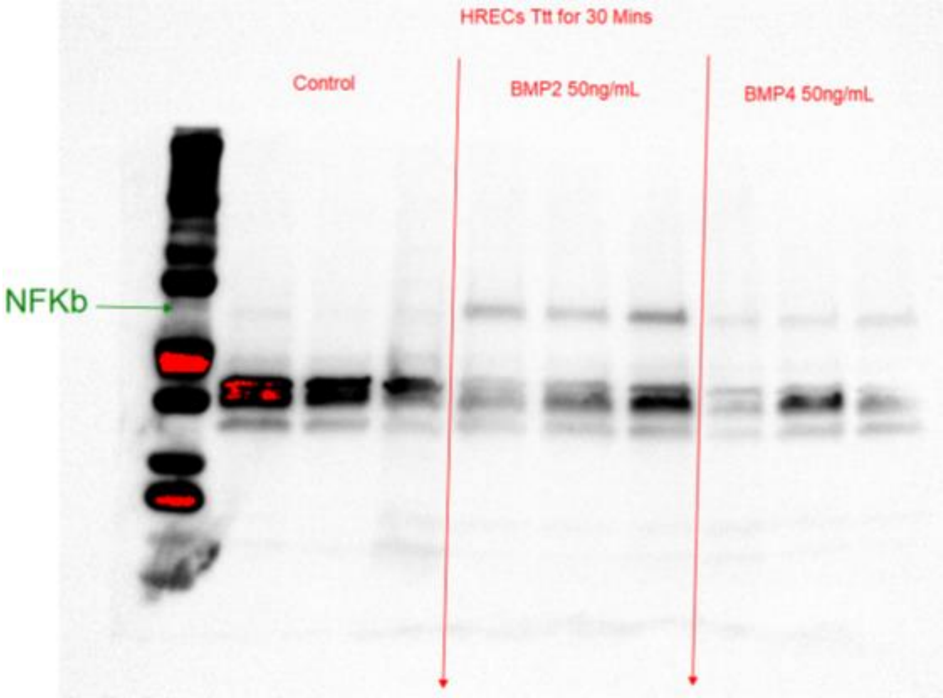
HRECs Ttt for 30 Mins
Control
BMP2 50ng/mL
BMP4 50ng/mL
NFKb

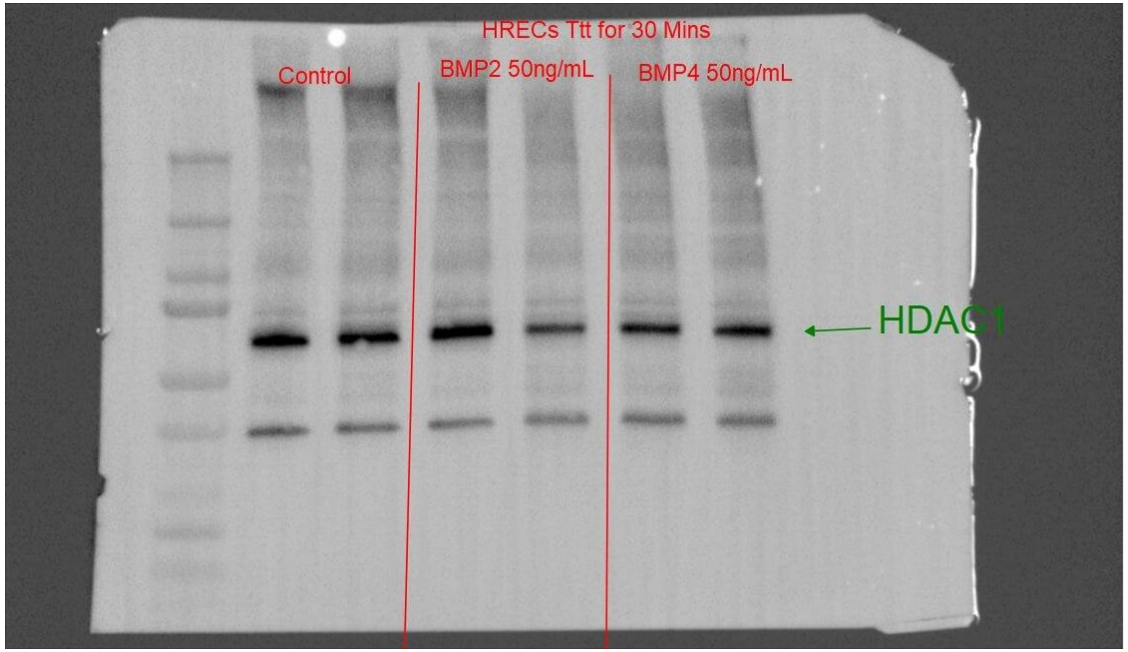
HRECs Ttt for 30 Mins
Control
BMP2 50ng/mL
BMP4 50ng/mL
HDAC1

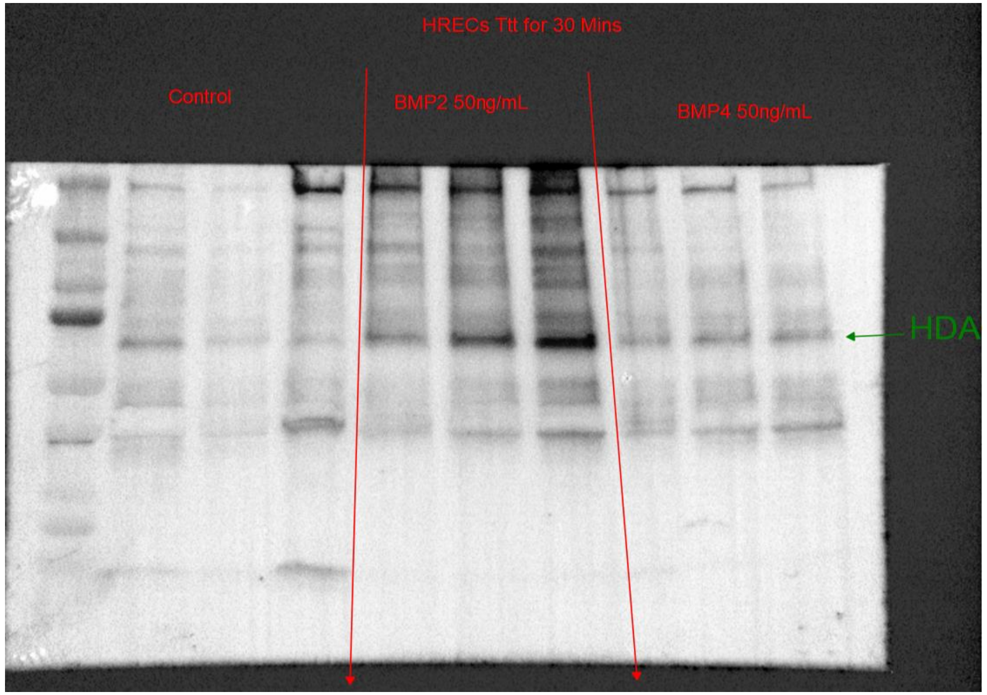
HRECs Ttt for 30 Mins
Control
BMP2 50ng/mL
BMP4 50ng/mL
HDA
